# CDT1 Is a Novel Prognostic and Predictive Biomarkers for Hepatocellular Carcinoma

**DOI:** 10.3389/fonc.2021.721644

**Published:** 2021-09-24

**Authors:** Chenhui Cai, Ying Zhang, Xu Hu, Wenhui Hu, Sizhen Yang, Hao Qiu, Tongwei Chu

**Affiliations:** ^1^ Department of Orthopedics, Xinqiao Hospital, Third Military Medical University (Army Medical University), Chongqing, China; ^2^ Department of Biomedical Materials Science, Third Military Medical University (Army Medical University), Chongqing, China

**Keywords:** CDT1, liver hepatocellular carcinoma, prognostic value, immune infiltration, bioinformatics analysis

## Abstract

**Objective:**

Hepatocellular carcinoma (HCC) is one of the most common malignant tumors endangering human health and life in the 21st century. Chromatin licensing and DNA replication factor 1 (CDT1) is an important regulator of DNA replication licensing, which is essential for initiation of DNA replication. CDT1 overexpression in several human cancers reportedly leads to abnormal cell replication, activates DNA damage checkpoints, and predisposes malignant transformation. However, the abnormal expression of CDT1 in HCC and its diagnostic and prognostic value remains to be elucidated.

**Methods:**

TCGA, ONCOMINE, UALCAN, HCCDB, HPA, Kaplan-Meier plotter, STRING, GEPIA, GeneMANIA, and TIMER were conducted for bioinformatics analysis. CDT1 protein expression was evaluated by immunohistochemistry in HCC tissues through a tissue microarray. qRT-PCR, western blot and a cohort of functional experiments were performed for *in vitro* validation.

**Results:**

In this study, we discovered remarkably upregulated transcription of CDT1 in HCC samples relative to normal liver samples through bioinformatic analysis, which was further verified in clinical tissue microarray samples and *in vitro* experiments. Moreover, the transcriptional level of CDT1 in HCC samples was positively associated with clinical parameters such as clinical tumor stage. Survival, logistic regression, and Cox regression analyses revealed the significant clinical prognostic value of CDT1 expression in HCC. The receiver operating characteristic curve and nomogram analysis results demonstrated the strong predictive ability of CDT1 in HCC. Kyoto Encyclopedia of Genes and Genomes and gene set enrichment analyses indicated that CDT1 was mainly associated with the cell cycle, DNA repair, and DNA replication. We further demonstrated the significant correlation between CDT1 and minichromosome maintenance (MCM) family genes, revealing abnormal expression and prognostic significance of MCMs in HCC. Immune infiltration analysis indicated that CDT1 was significantly associated with immune cell subsets and affected the survival of HCC patients. Finally, knockdown of CDT1 decreased, whereas overexpression of CDT1 promoted the proliferation, migration, invasion of HCC cells *in vitro*.

**Conclusions:**

Our study findings demonstrate the potential diagnostic and prognostic significance of CDT1 expression in HCC, and elucidate the potential molecular mechanism underlying its role in promoting the occurrence and development of liver cancer. These results may provide new opportunities and research paths for targeted therapies in HCC.

## Introduction

Hepatocellular carcinoma (HCC) is a serious disease with high morbidity and mortality, annually causing more than 500,000 deaths worldwide (1). HCC usually evolves from chronic liver inflammation, 80% of which is caused by viral hepatitis C or B (2). In the past decade, the prevalence and mortality of HCC have been decreasing in East Asia and other areas that traditionally report high incidence rates, while increasing in Europe and the United States (3). Although many researchers have delved into the biological and environmental mechanisms underlying liver cancer occurrence and progression, limited clinical options are currently available to delay or prolong tumor progression. Further, the high metastasis and recurrence rates of HCC pose significant challenges for diagnosis and treatment (4). Investigating the potential molecular mechanisms and effective prognostic signatures of HCC is thus urgently needed.

Maintaining the integrity of the genome requires strict and precise regulation of DNA replication, which needs to be coordinated with other cellular events to ensure that it only occurs once per cell cycle (5, 6). Chromatin licensing and DNA replication factor 1 (CDT1) is indispensable for the initiation of DNA replication, which plays a key role in eukaryotic cell replication and cell cycle regulation (7). The control of DNA initiation in the eukaryotic cell cycle requires coordination between multiple protein complexes. Initially, the origin recognition complex (ORC) directly binds to the site of DNA replication. ORC-DNA binding then recruits CDT1 and cell division cycle 6 (CDC6) to form a pre-replicating complex (pre-RC), which further loads minichromosome maintenance proteins (MCMs) onto chromatin (8). The cooperation of ORC, CDC6, CDT1, and MCMs at the initiation of replication ensures orderly DNA replication. Recent studies have reported that some cases of aberrant DNA replication and uncontrolled cell cycle progression may be attributable to dysregulated CDT1, and its destructive role has been identified in the tumor initiation, development, and chemoresistance of some tumor types (9, 10). Further, overexpression of CDT1 has been markedly associated with decreased survival and poor prognosis for some tumors (6, 11). Nevertheless, the prognostic significance and exact functions of CDT1 have not yet been determined in HCC progression.

**Figure 1:**
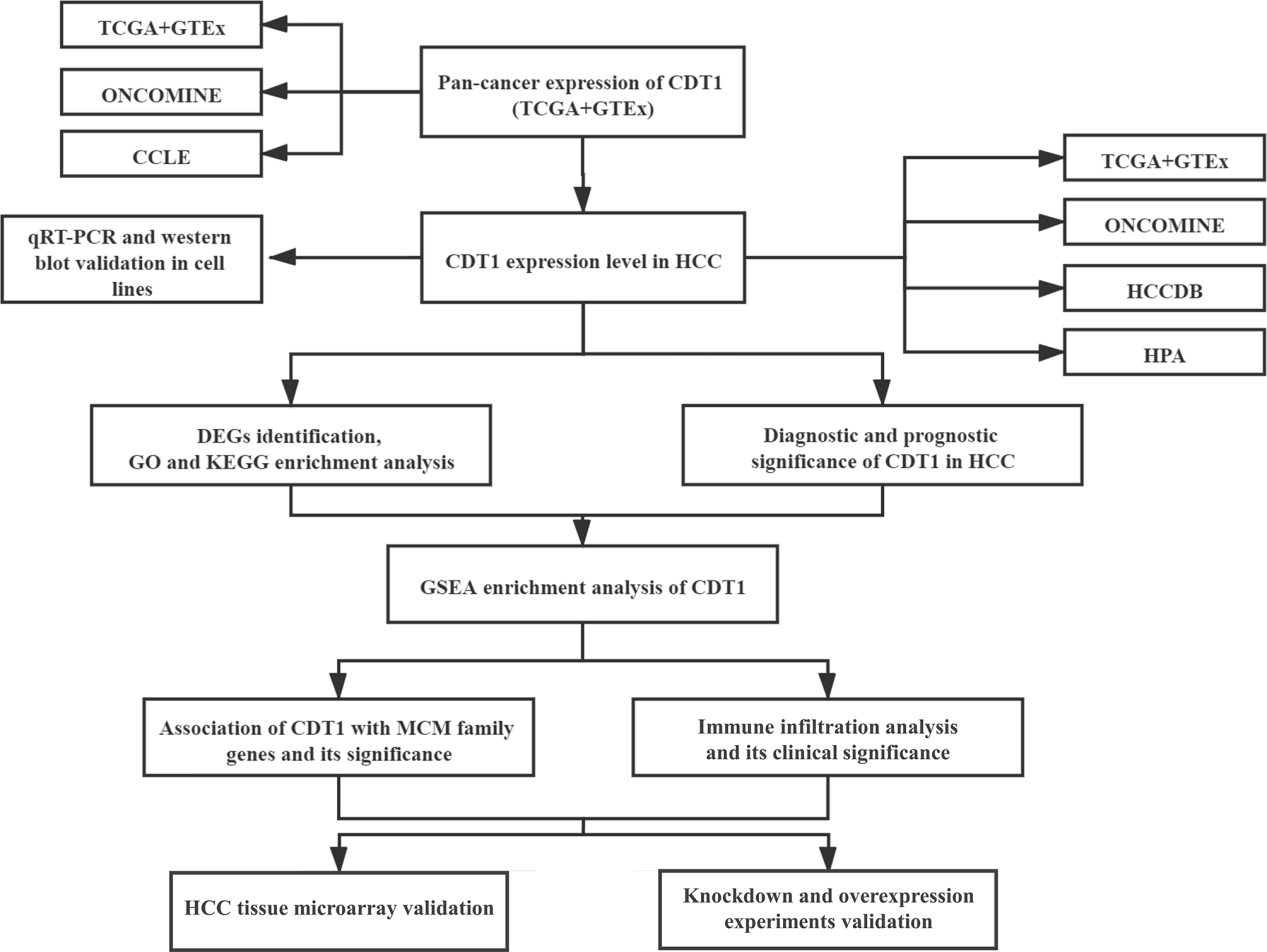
The workflow of the study.

Here, we aimed to comprehensively and systematically explore the expression of CDT1 in HCC through bioinformatics analysis, clinical tissue microarray samples and *in vitro* functional experiments using CDT1-knockdown and overexpression HCC cells. The study findings offer insight into the clinical significance, potential functions, interactive network, and association with immune infiltration of CDT1 in HCC, providing a novel prognostic biomarker for accurate survival prediction and precise targeted treatment of early-stage HCC. The workflow for this article is shown in **Figure 1**.

## Materials and Methods

### Data Resource

Level 3 gene expression profiles (level 3 data) were obtained from the liver hepatocellular carcinoma (LIHC) dataset in The Cancer Genome Atlas (TCGA) database (https://cancergenome.nih.gov/), comprised of 374 LIHC samples and 50 paracancerous tissues (Workflow Type: HTSeq-FPKM). HTSeq-FPKM values were then converted to TPM values (transcript per million) to compare differential expression among samples. Corresponding clinical information of HCC patients was also obtained from the TCGA data portal. A summary of clinical data is shown in **Supplementary Table 1**. For pan-cancer analysis, normal RNA-Seq data for 33 kinds of tumors were obtained from TCGA and Genotype-Tissue Expression (GTEx) samples using UCSC Xena (https://xenabrowser.net/).

### Comprehensive Analysis

ONCOMINE database (oncomine.org) is an integrated online data-mining tool, which provides an integrated analysis of genome-wide expression in multiple tumor samples and normal control samples (12). In our study, transcription levels of CDT1 in HCC samples and normal adjacent tissues were compared. Statistical significance was considered at *p* < 0.05, the fold change (FC) was set to 2, and the threshold for statistical significance was set at 10%.

The Cancer Cell Line Encyclopedia (CCLE) (www.broadinstitute.org/ccle) is a comprehensive portal that analyzes and visualizes genomic data from more than 1,000 tumor cell lines (13). Expression levels of CDT1 in multiple cancer cell lines were assessed by the CCLE dataset. HCCDB is a comprehensive visual database dedicated to the expression profile analysis of more than 3000 HCC samples (lifeome.net/database/hccdb/) (14). We utilized this powerful site to evaluate the expression of CDT1 in HCC. Besides, we analyzed the association between mRNA levels of CDT1 and survival outcomes in GSE14520 (HCCDB6) and ICGC-LIRI-JP (HCCDB18) datasets using the HCCDB database.

The Human Protein Atlas (HPA) is a publicly available source that provides immunohistochemical images for analyzing protein expression patterns in approximately 20 common tumors and normal tissues (https://www.proteinatlas.org) (15). Immunohistochemical images of clinical LIHC specimens and normal tissue samples were obtained from this database to compare CDT1 protein expression between the two groups.

UALCAN (http://ualcan.path.uab.edu/index.html) is a visual bioinformatics service platform that contains gene expression and clinicopathologic data from TCGA and MET500 cohort databases (http://ualcan.path.uab.edu/index.html) (16). In this study, we employed UALCAN to analyze correlations between mRNA expression of CDT1 and clinicopathological features. A p-value < 0.05 was considered significant.

Kaplan-Meier Plotter (https://kmplot.com/analysis/) is a comprehensive portal for analyzing the survival of cancer patients (17, 18), which was utilized to assess the prognostic significance of CDT1 in HCC by analyzing the association between mRNA levels of CDT1 and survival outcomes. Survival outcomes included overall survival (OS), progression-free survival (PFS), recurrence-free survival (RFS), and disease-specific survival (DSS). The optimal cutoff value was determined by the KM plotter algorithm. A p-value < 0.05 was considered significant.

### Cell Culture and Transfection

A normal human liver cell line (L-02 cells) and HCC cell lines (Hep3B, LM3, and SMMC-7721) were purchased from China Cell Bank (Shanghai, China). All cell lines were cultured in DMEM medium (Gibco, Waltham, MA, USA) with 10% fetal bovine serum (Ausbian, Australia) and 1% penicillin-streptomycin (Gibco). Cells were maintained in an incubator at a constant temperature of 37°C with 5% CO2. CDT1 siRNA oligonucleotides (40 nM) (5′-GCAUGUCAAGGAGCACCACAATT UUGUGGUGCUCCUUGACAUGCTT-3′), si-NC oligonucleotides (5′-UUCUCCGAACGUGUCACGUTTACGUGACACGUUCGGAGAATT-3′), CDT1 overexpression vector pEGFP-N1-CDT1 and empty control vector pEGFP-N1 were obtained from Shenggong Bioengineering Technology (Shanghai, China) and transfected into cells using Lipofectamine 3000 (Invitrogen, Waltham, MA, USA) according to the manufacturer’s instructions. CDT1 knockdown cells were obtained 72 h after transfection.

### RNA Extraction and Quantitative Real-Time PCR

Total RNA was obtained from cultured cells using Trizol reagent (Takara Bio, Kusatsu, Japan). SYBR Premix Ex Taq ™ II with a PCR detection system (Bio-Rad, Hercules, CA, USA) was applied to investigate the expression of indicated genes. cDNA was synthesized using the PrimeScript™ RT Reagent Kit (Takara Bio). Transcriptional levels were normalized against those of the internal control gene GAPDH. The following primers were used: GAPDH forward 5′-CCCAGCACAATGAAGATCAA-3′ and reverse 5′-ACATCGCTGGAAGGTGGAC-3′; CDT1 forward 5′-GACATGATGCGTAGGCGTTTT-3′ and reverse 5′-GAGCTGGTAATCTGACCTCCT-3′.

### Western Blotting

Cells were lysed and the protein concentration was determined by bicinchoninic acid assay. Proteins were subsequently separated by SDS-polyacrylamide gel electrophoresis and transferred to the polyvinylidene difluoride membrane. The membrane was blocked with 5% bovine serum albumin diluted with Tris-buffered saline with 0.1% Tween 20 (TBST) at room temperature for 2 h, and then incubated overnight at 4 °C with the primary antibodies anti-β-Tubulin (1:1000, Proteintech, Chicago, IL, USA) and anti-CDT1 (1:1000, Proteintech). The membrane was washed with TBST and incubated with secondary antibodies for 1.5 h (1:3000). The Enhanced Chemiluminescent detection kit (Biosharp, Beijing, China) was employed to visualize the protein bands.

### HCC Tissue Microarray and Immunohistochemical Staining

The human HCC tissue microarray (Cat No. IWLT-N-64LV41) was obtained from Wuhan Saiweier Biotechnology Co., Ltd. (Wuhan, China), including 14 HCC tissues samples and paired non-tumor tissues samples. IHC staining was carried out to measure CDT1 protein level in HCC tissues per the instructions from the manufacturer using an anti-CDT1 antibody (1:500, Proteintech). Each HCC sample was evaluated based on the staining intensity and the percentage of cells with positive staining. The H-score was calculated as (percentage of weak intensity cells ×1) + (percentage of moderate intensity cells ×2) + (percentage of strong intensity cells ×3). The numbers 0, 1, 2, 3 indicate the classification of positive cells. The H-score value ranges between 0 and 300. Paired t-test was used to compare the CDT1 expression in HCC tissues and the paired non-tumor tissues.

### Cell Proliferation, Invasion, and Migration Assays

Cell proliferation was assessed indirectly using Cell Counting Kit 8 (CCK‐8) and colony formation assays. For the CCK-8 assay, 10 μL aliquots of CCK-8 solution (Dojindo Laboratories, Kumamoto, Japan) were added to the wells of a 96-well plate, each well containing 2500 cancer cells. After incubation at 37°C for 1 h, the absorbance value at 450 nm was determined. For the colony formation experiment, 3000 cancer cells were seeded into six-well plates and the culture medium was changed every other day. The colonies were immobilized with paraldehyde and stained with crystal violet.

Cell migration and invasion ability were investigated using wound healing and Transwell assays, respectively. For the wound healing assay, a 200 μL pipette tip was used to make a single wound in each well when the confluence of transfected cells reached 90% in the six-well plate. Cell migration distance was calculated after incubation for 72 h in serum-free medium. The migration assay was performed in an 8 μm Transwell chamber (Corning, Rochester, NY, USA). The transfected HCC cells were inoculated on Matrigel containing serum-free medium at a density of 6 × 103 cells. The lower chamber was supplemented with a 400 μL medium containing 10% fetal bovine serum. Invasive cells were stained with 0.5% crystal violet.

### Screening of DEGs

Differentially expressed genes (DEGs) between HCC samples with high CDT1 expression (CDT1^high^) and low CDT1 expression (CDT1^low^) were identified using the DESeq2 package (19) in R (version 3.6.3) with thresholds of |logFC| > 0.5 and adjusted *p* < 0.05. Volcano plots and correlation heatmaps of differentially expressed mRNAs were constructed using the ggplot2 package in R.

### Functional Enrichment Analysis

To identify gene ontology (GO) annotations and Kyoto Encyclopedia of Genes and Genomes (KEGG) pathways in which CDT1 and its related DEGs were enriched. Besides, through the “HCC meta co-expression network” function of HCCDB database, we obtained genes with similar expression patterns to CDT1 in HCC and conducted further enrichment analysis of these co-expression genes. Functional enrichment analysis was conducted by using the clusterProfiler package in R (version 3.6.3) (20). Gene set enrichment analysis (GSEA) uses genome-wide expression profiling microarray data to compare gene enrichment to a predefined gene set (21). Gene expression data were divided into two groups according to CDT1 expression level: CDT1^high^ and CDT1^low^. The number of gene set permutations was set to 1000. Significant enrichment was defined as a gene set with a normal p-value < 5% and a false discovery rate of less than 25%.

### Interaction Analysis

Through detection of similar gene functions in GEPIA (http://gepia2021.cancer-pku.cn/) (22), we identified genes whose expression patterns were similar to that of CDT1 in HCC patients. String (https://string-db.org/) is a search tool that predicts the interactive network of genes and proteins (23). A protein-protein interaction (PPI) network analysis of CDT1 and its 30 most similar genes was conducted using STRING (an interaction score >0.7 was set as a cut-off criterion) and further processed using the visualization tool Cytoscape. GeneMANIA (genemania.org) is a visual database tool with highly accurate prediction algorithms, which provides information on physical interactions, co-expression, genetic interactions, and co-localization of query genes (24). We used GeneMANIA to construct a composite gene-gene functional interaction network of CDT1 and its 30 most similar genes.

### Immune Cell Infiltrates Analysis

Quantification of the infiltration level of 24 tumor-infiltrating immune cells in HCC samples was achieved by applying the ssGSEA method using the GSVA package in R. We scored the relative enrichment of every immunocyte type based on 509 gene signatures unique to 24 tumor-infiltrating lymphocytes, including B cells, T cells, macrophages, and neutrophils (25). Spearman correlation analysis was employed to evaluate the correlation between CDT1 expression and the level of immune cell infiltration, and the Wilcoxon rank-sum test was used to analyze the immune cell abundance in different CDT1 expression groups. TIMER (https://cistrome.shinyapps.io/timer/) is a publicly available portal tool to systematically analyze the infiltration of various immune subclasses and their effect on clinical outcomes (26). The “Survival module” in TIMER was used to evaluate correlations between clinical outcomes and infiltration level of immune cells.

### Statistical Analysis

Student’s t-test or one-way analysis of variance (ANOVA) was performed to analyze the statistical difference. Kaplan-Meier analysis was employed for evaluating patient survival. Survival difference was evaluated using Log-rank (Mantel-Cox) test. Univariate and multivariate Cox analyses were employed to evaluate the independent prognostic significance of CDT1 expression level and other clinical parameters on OS and DSS in HCC patients. Receiver operating characteristic (ROC) curves were established to evaluate the diagnostic significance of CDT1 expression using the pROC package in R (27), and the area under the ROC curve (AUC) indicated the magnitude of diagnostic efficiency. AUC > 0.7 and 0.5–0.7 indicated good accuracy and weak accuracy, respectively. Based on the expression values of CDT1 and other clinical parameters, we established a nomogram to predict OS of HCC patients at 1, 3, and 5 years. Spearman’s correlation coefficients were calculated to investigate the association between CDT1 and MCM family genes. All statistical analyses were conducted using R software (version 3.6.3). Statistical significance was defined as *p* < 0.05. Continuous data were presented as means ± standard deviation.

## Results

### Pan-Cancer Analysis of CDT1 Expression

**Figure 2:**
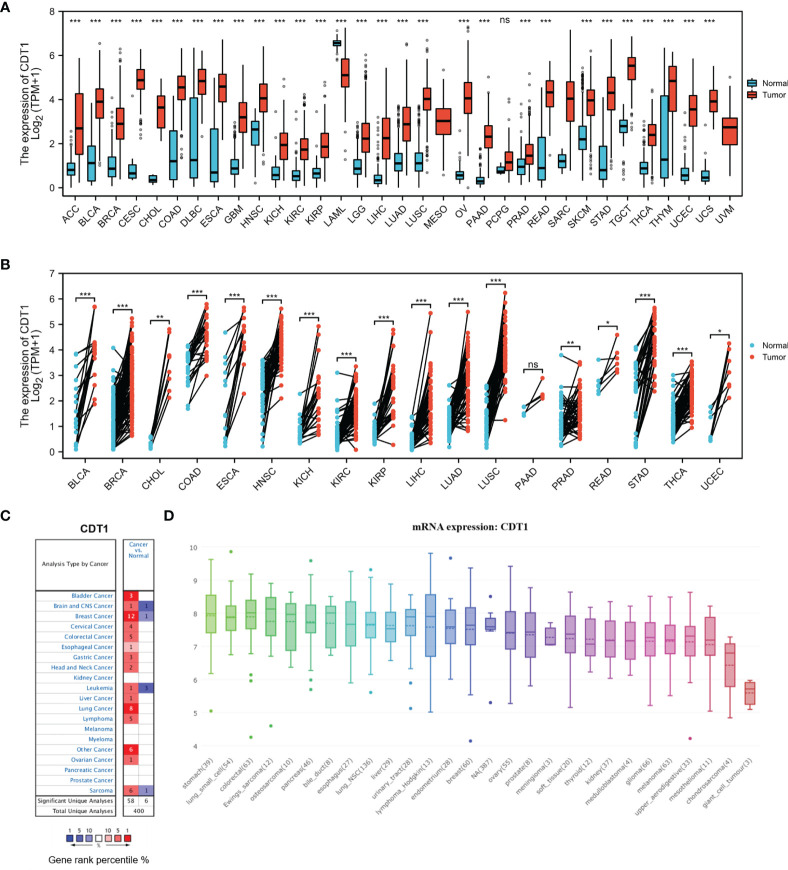
CDT1 expression levels in different types of human cancers. **(A)** Transcription expression of CDT1 in 33 distinct cancer types (TCGA and GTEx). **(B)** CDT1 expression in tumor and paired tissues (TCGA and GTEx). **(C)** Transcription expression of CDT1 in 20 distinct cancer types (ONCOMINE). **(D)** Expression of CDT1 in various tumor cell lines (CCLE). *p < 0.05, **p < 0.01, ***p < 0.001. The “ns” stands for not significant.

We examined CDT1 expression levels in different types of cancer using independent datasets from different sources. First, the transcriptional levels of CDT1 in various human cancers and their counterpart normal tissues were investigated in the TCGA and GTEx datasets. CDT1 expression was significantly higher in tumor tissues than in normal tissues for multiple cancers, including breast invasive carcinoma and stomach adenocarcinoma (**Figures 2A** **, B**). We then evaluated pan-carcinoma CDT1 expression levels using ONCOMINE, revealing the same expression trend as above (**Figure 2C**). Further, analysis of CDT1 expression levels in multiple common cancer cell lines from the CCLE database indicated that liver cancer cells had relatively higher CDT1 expression than other tumor cells (**Figure 2D**).

### CDT1 Expression in Hepatocellular Carcinoma From Different Databases

**Figure 3:**
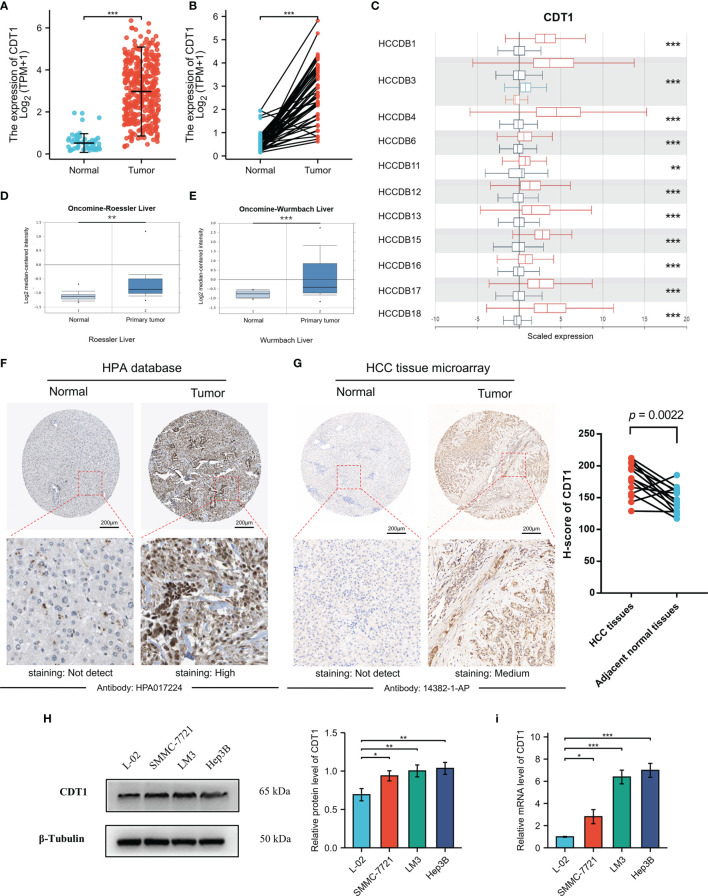
The relative expression of CDT1 in HCC at the cell and tissue levels. **(A)** CDT1 expression in normal and tumor tissues (TCGA and GTEx). **(B)** CDT1 expression in paired tissues (TCGA and GTEx). **(C)** The mRNA level of CDT1 in different HCC datasets (HCCDB). **(D, E)** CDT1 mRNA expression in normal and tumor tissues (ONCOMINE). **(F)** The protein expression level of CDT1 in HCC and normal liver tissues (HPA). **(G)** The IHC staining results of CDT1 level in HCC and adjacent non-tumor tissue (clinical tissue microarray of HCC) and the corresponding scatter diagram. Statistical significance was determined by paired t-test. **(H, I)** Western blot and qRT-PCR results of CDT1 expression in normal human liver cell line and HCC cell lines. Data are expressed as means ± SD. *p < 0.05, **p < 0.01, ***p < 0.001.

Although accumulating evidence suggests that CDT1 is a novel tumor biomarker (28, 29), transcriptional analysis of CDT1 in human HCC has not been well documented. Therefore, we utilized the TCGA data to compare transcriptional levels of CDT1 between HCC cancer samples and normal samples. mRNA expression levels of CDT1 were significantly increased in HCC samples relative to normal liver samples (*p* < 0.001) (**Figure 3A**). This conclusion was also verified in paired HCC and normal tissues (*p* < 0.001) (**Figure 3B**). We also compared transcriptional levels of CDT1 between HCC samples and normal control samples in the HCCDB dataset, which suggested abnormally high CDT1 expression in HCC (**Figure 3C**). The same conclusion was further confirmed in the ONCOMINE data (*p* < 0.001). Specifically, the Roessler and Wurmbach datasets indicated that CDT1 was upregulated in HCC tissues relative to normal tissues, with FCs of 1.285–1.852 (**Figures 3D** **, E**). Furthermore, high CDT1 protein expression was observed in HCC tissues based on the HPA dataset (**Figure 3F**). Besides, our IHC staining results on HCC tissue microarray demonstrated that CDT1 expression in HCC tissues was significantly higher than that in paired adjacent non-tumor tissues. The results of the paired scatter plot using paired t-test are shown in **Figure 3G**. Finally, the difference in CDT1 expression between normal and HCC cells was validated by qRT-PCR and western blot analysis of a normal human liver cell line and three HCC cell lines (**Figures 3H** **, I**).

### Relationship of mRNA Levels of CDT1 and Clinicopathological Features of HCC Patients

**Figure 4:**
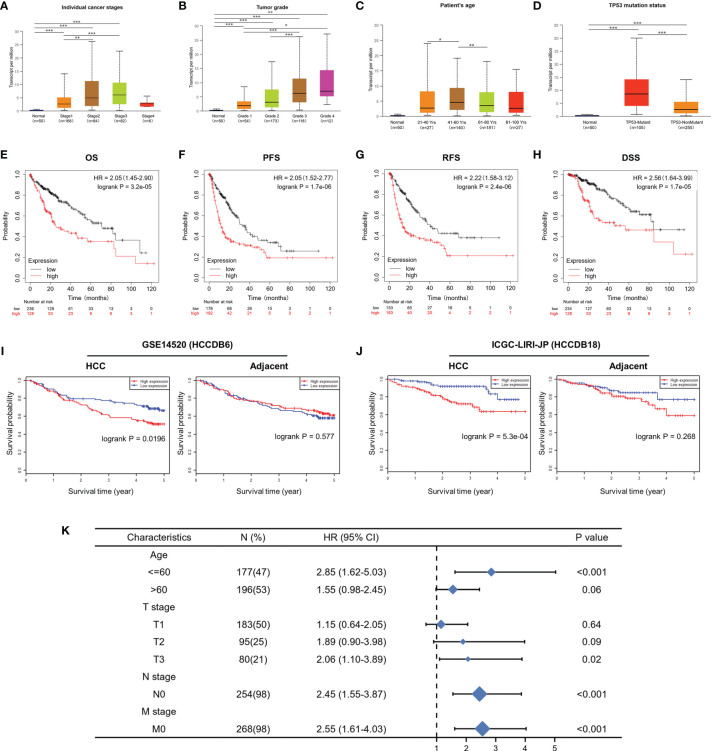
Correlation between CDT1 expression and the clinical parameters of HCC patients and its prognostic significance. **(A–D)** Relationship of CDT1 mRNA levels with individual cancer stages, tumor grade, age, and TP53 mutation status of HCC patients. **(E–H)** Relationship of CDT1 expression with OS, PFS, RFS, and DSS in TCGA database. **(I, J)** Relationship of CDT1 expression with OS of HCC patients in GEO and ICGC databases. **(K)** Forest plot showing the impact of CDT1 on OS at different TNM stages and ages. *p < 0.05, **p < 0.01, ***p < 0.001.

UALCAN was used to assess the relationship between CDT1 expression and the clinicopathologic features of HCC patients, including clinical cancer stage, pathological tumor grade, patient age, and TP53-mutation status. Higher levels of CDT1 mRNA tended to be expressed in tissues obtained from HCC patients with advanced cancer stages (p < 0.01). The highest mRNA levels of CDT1 were predominantly found in patients in stages II and III (**Figure 4A**). Pathological tumor grading has important prognostic significance. According to pathological tumor grading criteria, patients with high-grade tumors tended to exhibit higher mRNA levels of CDT1 (*p* < 0.05) (**Figure 4B**). Significantly high CDT1 expression was found mainly in the 41–60 year age group (*p* < 0.05) (**Figure 4C**). The p53 variation reportedly plays an important role in the occurrence and development of tumors (30). As expected, significant differences in CDT1 expression were identified between the TP53-mutation group and the normal and TP53-nonmutation groups (*p* < 0.001) (**Figure 4D**). Moreover, CDT1 expression was significantly associated with gender, race, pathologic stage, T stage, and alpha-fetoprotein level (*p* < 0.05) (**Supplementary Table 1**). Logistic regression analysis demonstrated that CDT1 expression was closely associated with a variety of clinical characteristics of poor prognosis such as pathological stage (OR = 2.304, 95% confidence interval [CI] = 1.505-3.548, *p* < 0.001), T stage (OR = 2.429, 95% CI = 1.605–3.699, *p* < 0.001), alpha-fetoprotein (OR = 3.428, 95% CI = 1.908–6.361, p < 0.001), and histological grade (OR = 3.256, 95% CI = 2.095–5.123, *p* < 0.001) (**Table 1**). Furthermore, various survival parameters were also evaluated for their relationship with CDT1 mRNA levels in HCC patients. Survival analysis demonstrated that the OS (defined by period from suffering to death), PFS (reflecting tumor worsening), RFS (referring to time from primary treatment to recurrence), and DSS (reflecting death from cancer itself) rates of HCC patients with high CDT1 expression were significantly lower than those of patients with low CDT1 expression (*p* < 0.001) (**Figures 4E** **–H**). Further survival analysis was performed using the GSE14520 (HCCDB6) and ICGC-LIRI-JP (HCCDB18) datasets on HCCDB. The same conclusion was reached (**Figures 4I** **, J**). We also evaluated the impact of CDT1 expression on OS in HCC patients of different ages and TNM stages (**Figure 4K**). In addition, we assessed the independent prognostic value of CDT1 using Cox proportional hazards regression analysis based on the RNA-Seq data and clinical information from the TCGA dataset. The results demonstrated that a high transcriptional level of CDT1 was independently correlated with significantly shorter OS (HR = 1.588, 95% CI: 1.067–2.363, and *p* = 0.023) and DSS (HR= 2.609, 95% CI: 1.323–5.147, and *p* = 0.006) for HCC patients (**Table 2**). The transcriptional level of CDT1 was confirmed to be an independent prognostic factor for OS and DSS in HCC patients.

**Table 1:** Logistic analysis of the association between CDT1 expression and clinical characteristics in HCC patients.

Characteristics	Total (N)	Odds Ratio (OR)	*P*-value
Age (>60 *vs*. <=60)	373	0.748 (0.497-1.124)	0.163
**Gender** (Male *vs*. Female)	374	0.627 (0.403-0.969)	**0.036**
**Race** (Black or African American &White *vs*. Asian)	362	0.504 (0.330-0.767)	**0.001**
**Pathologic stage** (Stage II & Stage III & Stage IV *vs*. Stage I)	350	2.304 (1.505-3.548)	**<0.001**
T stage (T2&T3&T4 *vs*. T1)	371	2.429 (1.605-3.699)	**<0.001**
N stage (N1 *vs*. N0)	258	0.896 (0.106-7.557)	0.913
M stage (M1 *vs*. M0)	272	0.283 (0.014-2.240)	0.277
Residual tumor (R1&R2 *vs*. R0)	345	1.321 (0.508-3.540)	0.568
**AFP (ng/ml)** (>400 *vs*. <=400)	280	3.428 (1.908-6.361)	**<0.001**
Child-Pugh grade (B&C *vs*. A)	241	0.655 (0.253-1.594)	0.362
Fibrosis ishak score (3/4&5/6 *vs*. 0&1/2)	215	0.917 (0.533-1.576)	0.754
Vascular invasion (Yes *vs*. No)	318	1.510 (0.950-2.410)	0.082
**Histologic grade** (G3&G4 *vs*. G1&G2)	369	3.256 (2.095-5.123)	**<0.001**
Adjacent hepatic tissue inflammation (Mild&Severe *vs*. None)	237	1.485 (0.889-2.493)	0.132
Albumin(g/dl) (>=3.5 *vs*. <3.5)	300	1.076 (0.628-1.855)	0.790
**Prothrombin time** (>4 *vs*. <=4)	297	0.471 (0.278-0.784)	**0.004**

Bold values indicate significant p-values.

**Table 2:** Cox regression analyses of variables for OS and DSS in LIHC patients.

Characteristics	OS				DSS			
	Univariate analysis		Multivariate analysis		Univariate analysis		Multivariate analysis	
	Hazard ratio (95% CI)	*P* value	Hazard ratio (95% CI)	*P* value	Hazard ratio (95% CI)	*P* value	Hazard ratio (95% CI)	*P* value
Age (>60 *vs*. <=60)	1.205 (0.850-1.708)	0.295			0.846 (0.543-1.317)	0.458		
Gender (Male *vs*. Female)	0.793 (0.557-1.130)	0.200			0.813 (0.516-1.281)	0.373		
**Race (Non-Asian *vs*. Asian)**	1.341 (0.926-1.942)	0.121			1.500 (0.927-2.427)	0.098	2.671 (1.183-6.030)	**0.018**
**Pathologic stage (Stage II& III& IV *vs*. I)**	2.090 (1.429-3.055)	**<0.001**	1.772 (1.180-2.661)	**0.006**	2.909 (1.718-4.925)	**<0.001**	1.951 (0.973-3.914)	0.060
Residual tumor (R1&R2 *vs*. R0)	1.604 (0.812-3.169)	0.174			1.678 (0.728-3.870)	0.224		
AFP (ng/ml) (>400 *vs*. <=400)	1.075 (0.658-1.759)	0.772			0.867 (0.450-1.668)	0.668		
**Child-Pugh grade (B&C *vs*. A)**	1.643 (0.811-3.330)	0.168			2.560 (1.123-5.834)	**0.025**	2.848 (1.195-6.788)	**0.018**
Fibrosis ishak score (3/4&5/6 *vs*. 0&1/2)	0.740 (0.445-1.232)	0.247			0.660 (0.340-1.279)	0.218		
Vascular invasion (Yes *vs*. No)	1.344 (0.887-2.035)	0.163			1.277 (0.707-2.306)	0.418		
**Tumor status (With tumor *vs*. Tumor free)**	2.317 (1.590-3.376)	**<0.001**	1.809 (1.210-2.703)	**0.004**	2.389 (1.760-2.872)	0.094		
**CDT1 (High *vs*. Low)**	1.916 (1.347-2.724)	**<0.001**	1.588 (1.067-2.363)	**0.023**	2.297 (1.452-3.634)	**<0.001**	2.609 (1.323-5.147)	**0.006**

Bold values indicate significant p-values.

### Diagnostic Value of CDT1 Expression in HCC

**Figure 5:**
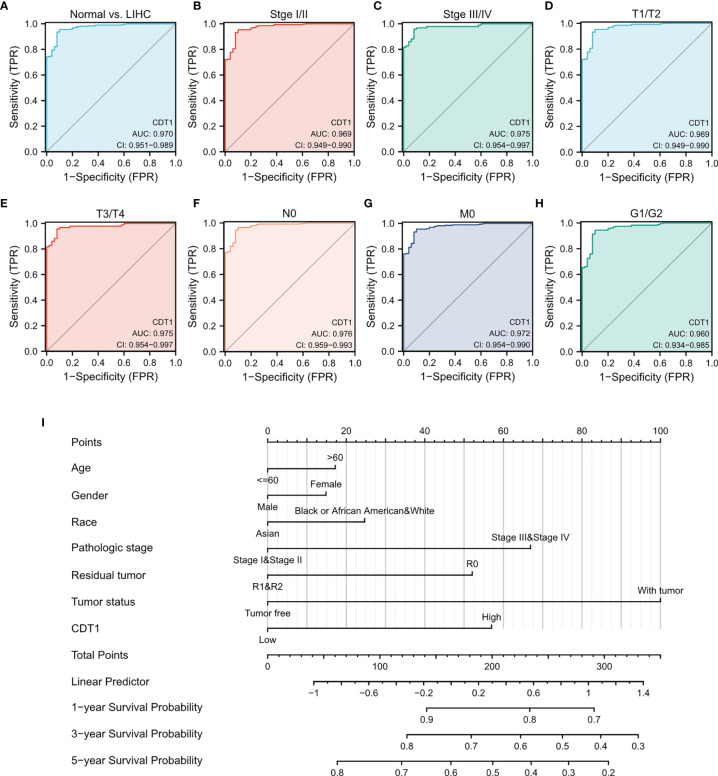
Diagnostic value of CDT1 mRNA level in HCC. **(A)** ROC curve for CDT1 in HCC and normal liver tissue. **(B–H)** Subgroup analysis for stage I/II, stage III/IV, T1/T2, T3/T4, N0, M0, and G1/G2. **(I)** Nomogram predicting the probability of patients with 1-, 3- and 5-year overall survival.

The ROC curve analysis demonstrated the strong value of CDT1 in the diagnosis of HCC (**Figure 5A** **)**.

Next, we evaluated the diagnostic value of CDT1 expression for different clinical features of HCC patients. Specifically, the AUC values were 0.969 for stage I/II, 0.975 for stage III/IV, 0.969 for stage T1/T2, 0.975 for stage T3/T4, 0.976 for stage N0, 0.972 for stage M0, and 0.960 for stage G1/G2 (**Figures 5B** **–H**). Furthermore, we established a nomogram combining CDT1 expression and key clinical factors to predict the 1-, 3-, and 5-year survival of HCC patients. A higher nomogram score for OS indicated a worse prognosis (**Figure 5I**). These results implied that the transcriptional level of CDT1 was relatively sensitive and specific for the diagnosis of HCC.

### Identification of Differentially Expressed Genes

**Figure 6:**
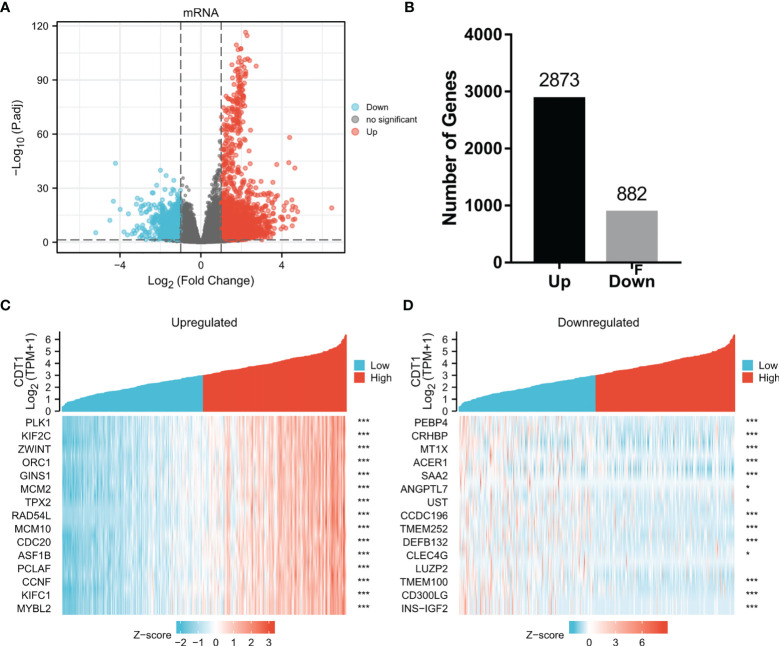
Volcano plots and heatmap plots of DEGs between the expression of CDT1^high^ and CDT1^low^ in HCC samples. **(A)** The volcano plot described 3755 DEGs (|log2fold change| > 1 and adjusted *p*-value < 0.05). **(B)** The histogram showing the number of up-regulated or down-regulated genes. **(C, D)** The heatmaps depicted the expression of 15 significant upregulated and downregulated genes in HCC samples with CDT1^high^ and CDT1^low^ expression. *p < 0.05, ***p < 0.001.

To explore the abnormal changes in downstream pathways caused by high expression of CDT1, we identified DEGs between HCC samples with CDT1high and CDT1low mRNA expression based on the TCGA data. Among a total of 3755 DEGs, 2873 were upregulated and 882 were downregulated. Volcano plots and bar graphs were generated to visually display the distribution of DEGs (**Figures 6A** **, B**), and heatmaps depicted the top 15 significantly upregulated and downregulated DEGs between the CDT1high and CDT1low expression groups (**Figures 6C** **, D**).

### Enrichment Analysis of CDT1 and Their Most Similar Genes

**Figure 7:**
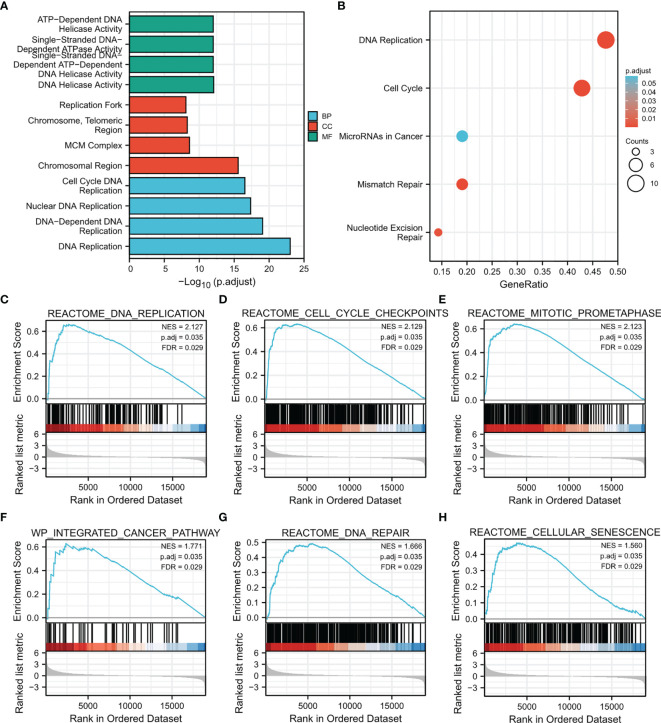
Results of enrichment analysis. **(A, B)** GO and KEGG enrichment results of CDT1-related DEGs (15 most significant upregulated and 15 most significant downregulated genes in HCC samples with CDT1^high^ and CDT1^low^ expression). **(C–H)** Gene set enrichment plots of **(C)** DNA replication, **(D)** cell cycle checkpoints, **(E)** mitotic prometaphase, **(F)** integrated cancer pathway, **(G)** DNA repair, and **(H)** cellular senescence with high CDT1 expression from GSEA.

To further clarify the potential mechanisms of CDT1 in HCC progression, GO and KEGG enrichment was performed to predict the functions and pathways of the top 15 upregulated and downregulated CDT1-related DEGs. The biological processes for these genes were predominantly enriched in DNA replication initiation, nuclear division, mitotic nuclear division, and organelle fission. The molecular functions for these genes mainly included DNA replication origin binding, 3’-5’ DNA helicase activity, helicase activity, and ATPase activity. The CDT1-related DEGs were mainly enriched in the chromosomal region, condensed chromosome kinetochore, and spindle in terms of the cellular component category (**Figure 7A**). The results of KEGG enrichment revealed several major pathways: cell cycle, DNA replication, and homologous recombination (**Figure 7B**). Besides, through the “HCC meta co-expression network” function of the HCCDB database, we acquired genes with similar expression patterns to CDT1 in HCC and conducted further enrichment analysis of these co-expression genes (MCM2, MCM10, CDCA5, CDC45, TEDC2, GINS2, etc.) (**Supplementary Figure 1A**). The GO and KEGG enrichment results of CDT1-related co-expression genes were similar as described above (**Supplementary Figures 1B, C**). The CDT1-related DEGs were further analyzed using GSEA to identify signaling pathways that were significantly enriched (FDR < 0.25, adjusted p-value < 0.05) in HCC. Based on normalized enrichment scores, DNA replication, DNA repair, prometaphase mitosis, cell senescence, and pathway in cancer were significantly enriched (**Figures 7C** **–H**). All the above-enriched pathways were markedly associated with the occurrence and progression of malignant tumors.

### Molecular Interactions of CDT1 in HCC

**Figure 8:**
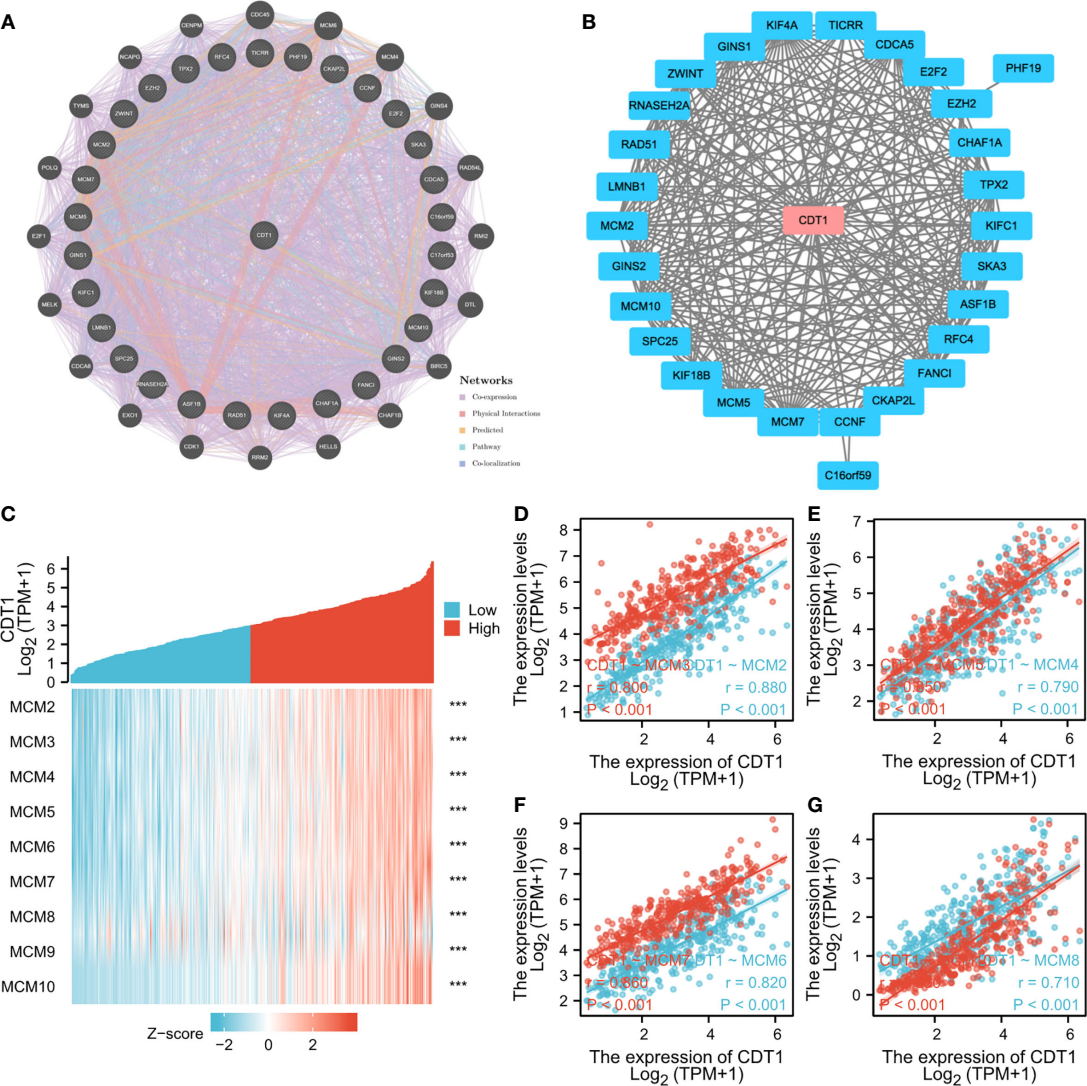
**(A, B)** Interaction network of CDT1 and their most similar genes on GeneMANIA **(A)** and STRING **(B)** datasets. **(C)** Heatmap of the expression of MCM family genes in HCC samples with CDT1^high^ and CDT1^low^ expression. **(D–G)** Scatter plot results using Spearman correlation analysis between MCMs and CDT1 at the transcriptional level. ***p < 0.001.

**Figure 9:**
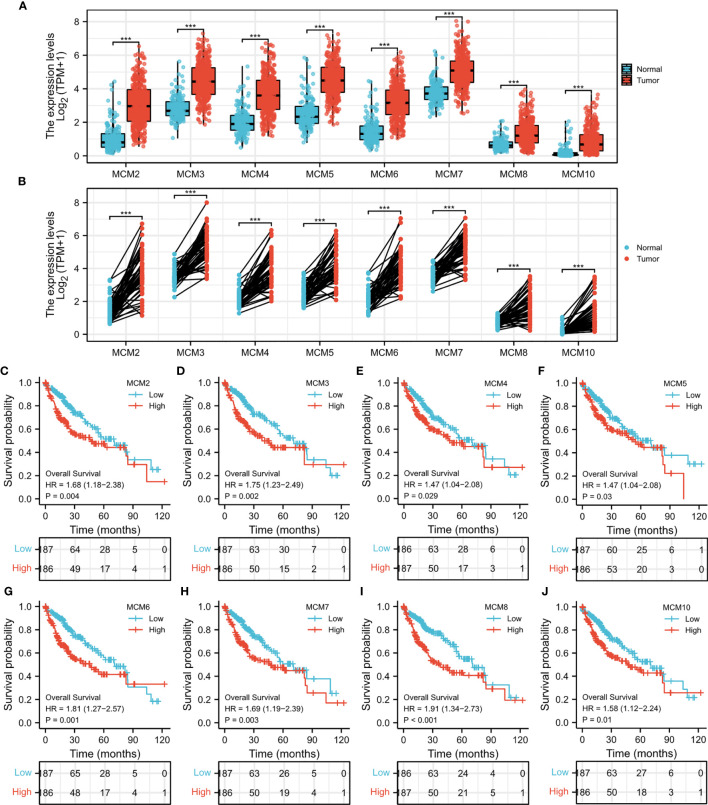
**(A)** mRNA expression levels of all MCM family genes in HCC tissues and normal tissues. **(B)** The expression of MCM family members in paired HCC tissues. **(C–J)** Survival analysis of MCMs in HCC. ***p < 0.001.

Through detection of similar gene functions in GEPIA, we identified genes whose expression patterns were similar to CDT1 in HCC patients. We constructed a PPI network to elucidate the potential interactions between CDT1 and genes with similar functions (**Figure 8B**). CDT1 and genes similar to it (e.g., KIFC1, RNASEH2A, MCM2, E2F2, and CCNF) were associated with DNA replication origin binding, helicase activity, DNA binding, nucleic acid-binding, and MCM complex. We also constructed a PPI network to explore the interactions between CDT1 and its co-expressing genes. Similar to the above results, the interaction network of CDT1-related co-expressed genes is mainly associated with cell cycle and DNA replication (**Supplementary Figure 2**). Besides, the gene-gene interaction network also confirmed that CDT1 and its associated genes were primarily associated with DNA replication, DNA-dependent DNA replication, DNA strand elongation, and MCM complex (**Figure 8A**). From the results of our interaction analyses, we identified a correlation between CDT1 and MCM family genes in HCC. It is now widely accepted that CDT1 cooperates with CDC6 to load MCMs onto the ORC and further induce chromatin unfolding (31). Therefore, we further analyzed the correlation between MCMs and CDT1. The heatmap depicted the expression of MCM family genes in HCC samples with CDT1high and CDT1 low expression (**Figure 8C**). The Scatter plot obtained using Spearman correlation analysis indicated that MCMs, except MCM9, were highly correlated with CDT1 at the transcriptional level (**Figures 8D–G** and **Supplementary Figure 3A**). We further utilized the TCGA data to compare transcriptional levels of MCMs between HCC cancer samples and normal samples. The results indicated that mRNA expression levels of all MCM family genes, except MCM9, were significantly higher in HCC tissues than normal tissues and paired tissues (*p* < 0.001) (**Figures 9A, B** and **Supplementary Figure 3B**). Furthermore, high expression of MCMs, except MCM9, was significantly associated with shorter OS (*p* < 0.001) (**Figures 9C–J** and **Supplementary Figure 3C** **)**. To sum up, our present study indicated a close relationship of MCMs with CDT1 as well as their expression and prognostic significance in HCC patients, which suggests the potential mechanism by which CDT1 and MCM family genes cooperate to promote the occurrence and development of HCC.

### Correlation Analysis Between CDT1 Expression and Various Immune Infiltrates

**Figure 10:**
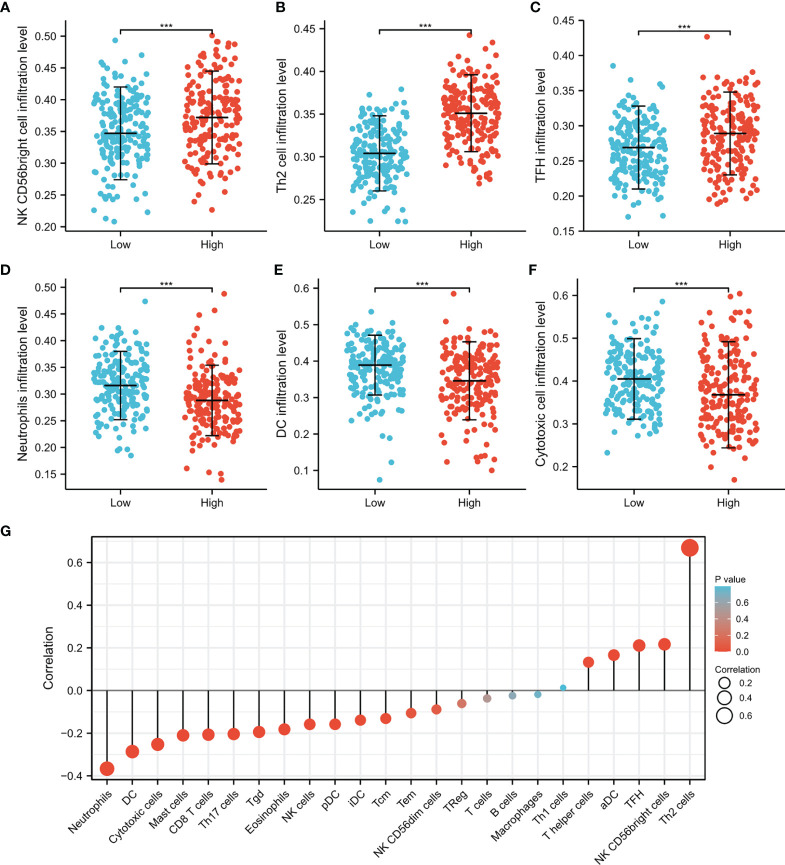
The CDT1 expression and immune cell infiltration using the TIMER database. **(A–F)** Different proportions of immune cell subtypes in HCC samples in CDT1^high^ and CDT1^low^ groups. **(G)** The correlation between CDT1 expression and 24 immune infiltrating cells. ***p < 0.001.

Immune cells in the tumor microenvironment largely influence the biological behavior of the tumor (32, 33). Investigating infiltration of various immune cells in the HCC micro-environment, we demonstrated that CDT1 expression was positively correlated with the abundance of immunocytes such as T helper 2 (Th2) cells, activated dendritic cells, and T follicular helper cells, but was negatively correlated with the abundance of innate immunocytes such as neutrophils, dendritic cells, cytotoxic cells, and mast cells (**Figures 10A** **–G**). Moreover, we assessed the independent prognostic value of immune cell infiltration and CDT1 expression using Cox proportional hazards regression analysis using TIMER. The results indicated that the expression of CDT1 and the degree of infiltration of all six immune cells, except neutrophils, were independently associated with significantly shorter OS (**Table 3**).

**Table 3:** The Cox proportional hazard model of CDT1 and six tumor-infiltrating immune cells in HCC.

Characteristics	Coef	HR	95%CI_l	95%CI_u	*p*-value	Sig
Age	0.014	1.014	0.997	1.032	0.108	
Gender Male	-0.029	0.972	0.604	1.563	0.906	
**Race Black**	1.134	3.109	1.098	8.805	0.033	*
Race White	-0.027	0.973	0.585	1.619	0.917	
Stage2	0.144	1.155	0.676	1.972	0.598	
**Stage3**	0.659	1.934	1.178	3.174	**0.009**	**
**Stage4**	1.522	4.583	1.314	15.984	**0.017**	*
Purity	0.447	1.564	0.481	5.087	0.458	
**B cell**	-7.993	0.000	0.000	0.625	**0.037**	*
**CD8+T cell**	-5.103	0.006	0.000	0.947	**0.048**	*
**CD4+T cell**	-7.616	0.000	0.000	0.85	**0.045**	*
**Macrophage**	8.351	4232.983	15.673	1143259	**0.003**	**
Neutrophil	-2.399	0.091	0.000	10284.16	0.686	
**Dendritic**	4.706	110.594	2.46	4972.626	**0.015**	*
**CDT1**	0.267	1.306	1.073	1.589	**0.008**	**

Bold values indicate significant p-values. *p < 0.05, **p < 0.01.

### CDT1 Knockdown Inhibited, Whereas Overexpression Promoted Tumorigenicity of HCC Cells *In Vitro*

**Figure 11:**
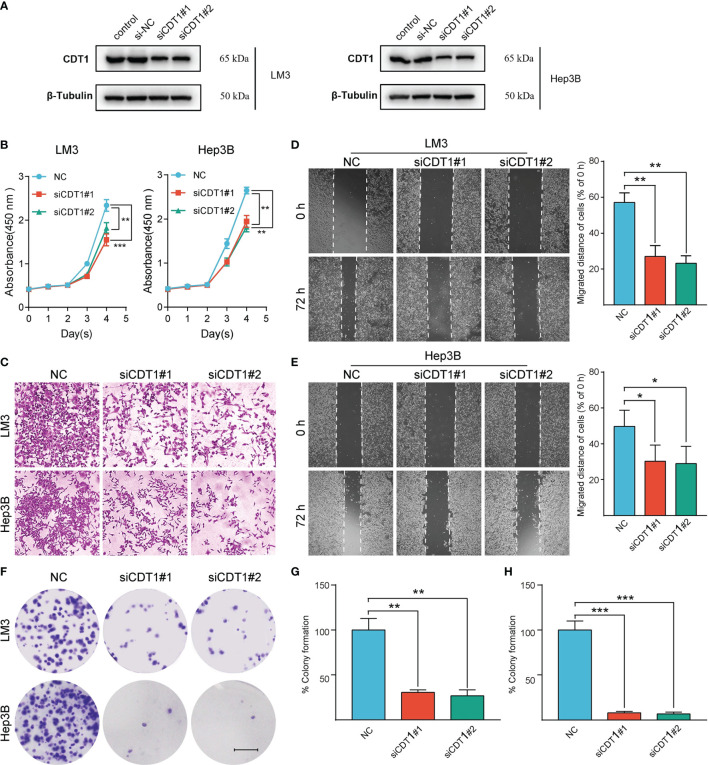
Silencing of CDT1 inhibited the proliferation, migration and invasion of HCC cells. **(A)** Western blot detection of CDT1 expression after knockdown of CDT1 in liver cancer cells. **(B)** The effect of CDT1 knockdown on cell viability at 24, 48, 72 and 96h after seeding in plates was measured by CCK-8 assay. **(C–E)** Transwell analysis and wound healing assay reflected the migration ability of LM3 and Hep3B cell lines. **(F)** Images of the colony formation assay after knockdown of CDT1 in HCC cells **(G, H)** Relative quantification of the colony areas is shown. Scale bars in **(F)** equal 5mm. Data are expressed as means ± SD of three independent experiments. *p < 0.05, **p < 0.01, ***p < 0.001.

**Figure 12:**
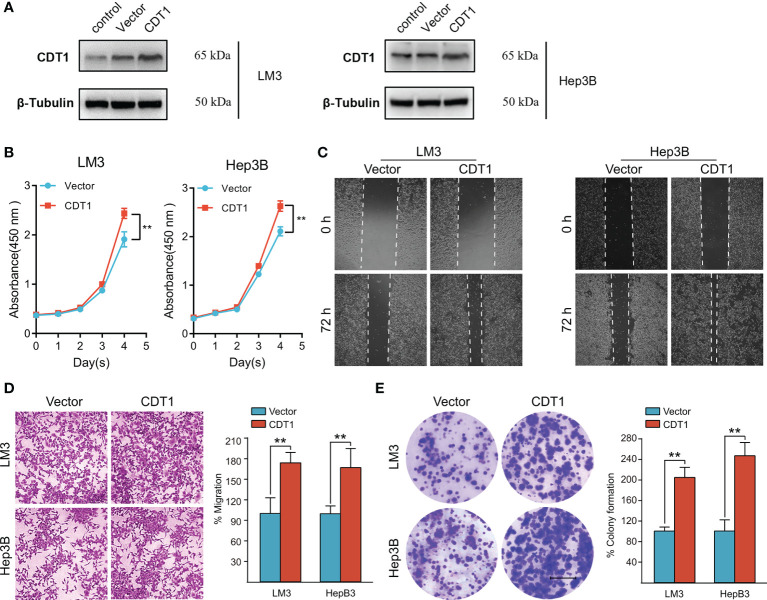
Overexpression of CDT1 promoted the proliferation, migration and invasion of HCC cells. **(A)** Western blotting detection of CDT1 expression after overexpression of CDT1 in the LM3 and Hep3B cell lines. **(B)** The effect of CDT1 overexpression on cell viability at 24, 48, 72 and 96h after seeding in plates was measured by CCK-8 assay. **(C, D)** Transwell analysis and wound healing assay reflected the migration ability of LM3 and Hep3B cell lines. **(E)** Images of the colony formation assay after overexpression of CDT1 in HCC cells. Scale bars in **(E)** equal 5mm. Data are expressed as means ± SD of three independent experiments.**p < 0.01.

We further validated the role of CDT1 in HCC *in vitro*. Since CDT1 expression in the LM3 and Hep3B cell lines was higher than that in other cell lines (**Figure 3H**), they were selected for functional analysis. The interference efficiency of CDT1 knockdown by siRNA was detected using western blotting (**Figure 11A**). The results of CCK-8 and colony formation assays demonstrated that CDT1 knockdown significantly inhibited the proliferation and invasion abilities of LM3 and Hep3B cells (**Figures 11B** **, F–H**). Further, the wound healing and transwell assays demonstrated that CDT1 knockdown significantly inhibited the migration of HCC cells (**Figures 11C** **–E**). The function of CDT1 was further investigated by performing overexpression studies. The efficiencies of CDT1 overexpression in LM3 and Hep3B cell lines were detected by western blotting (**Figure 12A**). Consistent with the knockdown experiments, the results of CCK-8, colony formation, wound healing and transwell assays showed that overexpression of CDT1 significantly promoted HCC cells proliferation, invasion, and migration (**Figures 12B** **–E**).

## Discussion

HCC is one of the most common cancers and the second leading cause of death among cancer patients, accounting for 600,000 deaths each year (1). Numerous studies have shown that metastasis at the later stage is the leading cause of HCC-related mortality, which highlights the importance of early diagnosis and disease management (34). Although landmark advances in HCC diagnosis have been achieved in recent years, only a small proportion of liver cancer cases are detected and diagnosed at an early stage (35). Moreover, despite recent breakthroughs in diagnosis and treatment, the prognosis of HCC patients remains far from satisfactory. Therefore, effective biomarkers and novel therapeutic targets for HCC are urgently needed.

A growing body of evidence has demonstrated that abnormal DNA replication and uncontrolled cell cycle progression are important hallmarks of tumor genesis, invasion, and progression (5). CDT1 is thought to participate in the coordination of the cell cycle and proliferation in eukaryotic cells by forming a pre-RC at the beginning of the cell cycle, which further loads MCMs onto chromatin (8). At present, some studies have reported that abnormally high expression of CDT1 is explicitly associated with the occurrence, development, and malignant behavior of tumors (6, 9, 10). However, the exact role of CDT1 proteins in HCC remains unknown. In our study, we systematically characterized CDT1 in HCC, revealing its expression profile, predictive and prognostic significance, potential functions, interactive network, miRNA regulation, and association with infiltration levels of immune subsets.

We first examined the transcriptional levels of CDT1 in different types of cancer using independent datasets from three different sources (ONCOMINE, TCGA, and GTEx). CDT1 was highly expressed in various tumors including cervical cancer, breast cancer, colorectal cancer, and liver cancer. Similarly, high CDT1 expression was identified in a variety of tumor cells in the CCLE database. Together, these results suggest that CDT1 may have a potential promoting role in tumor development.

Subsequently, we revealed significantly higher transcriptional levels of CDT1 in HCC specimens than in normal samples. Elevated CDT1 expression has been detected in several cancers, including lung cancer, breast cancer, and lymphoma (6, 10, 11). Karakaidos et al. examined a large number of non-small cell lung cancer samples and corresponding normal lung samples, reporting that CDT1 was overexpressed in most lung cancer tissues at mRNA and protein levels (10). Additionally, a recent study reported that the transcriptional level of CDT1 was significantly increased in breast cancer cells compared with normal breast epithelial cells (6). Further, high CDT1 expression has been associated with undesirable prognosis of breast cancer patients. Seo et al. provided strong evidence that CDT1 can promote tumor development *in vivo*. Using the Lck promoter element to overexpress CDT1 in T cells, these researchers reported the progression of p53-knockout lymphoblastic lymphoma (11). In the current study, higher transcriptional levels of CDT1 were identified in HCC samples compared to normal liver samples across a variety of databases. CDT1 protein expression in HCC tissues was also significantly higher than that in normal liver tissues in the HPA dataset and clinical tissue microarray. To further verify our conclusion, we detected the relative expression levels of CDT1 in various HCC cell lines and a normal liver cell line by qRT-PCR and western blotting, obtaining results that were consistent with our bioinformatics analysis.

We further investigated the relationship between CDT1 expression and the clinical characteristics of HCC patients, revealing that CDT1 expression was correlated with tumor stage and TP53-mutation status. Logistic regression analysis indicated that CDT1 expression was significantly associated with alpha-fetoprotein, pathologic stage, histologic grade, and other clinical parameters in HCC patients. The Kaplan-Meier test indicated that high CDT1 expression was suggestive of undesirable OS, RFS, DFS, and DSS prognoses of HCC patients. Univariate and multivariate regression analyses confirmed that high CDT1 expression was an independent adverse prognostic factor for OS and DSS in HCC. At present, a prediction profile of HCC based on CDT1 expression has not been reported, but our ROC curve analysis suggested that CDT1 expression has significant value in the diagnosis of HCC. We further established a nomogram by integrating various clinical parameters and CDT1 mRNA levels from the TCGA dataset to predict individual patient mortality risk and help optimize therapy decisions.

To explore the abnormal changes in downstream pathways caused by high CDT1 expression in HCC, we identified DEGs between HCC patients with high and low CDT1 expression. GO and KEGG enrichment results revealed that the above DEGs mainly participated in cell cycle, DNA replication, and 3’-5’ DNA helicase activity. Furthermore, GSEA analysis revealed that the DEGs were significantly enriched in cell cycle checkpoints, DNA repair, DNA replication, prometaphase mitosis, cell senescence, and pathway in cancer. All the above-enriched pathways were markedly correlated with the occurrence and progression of malignant tumors.

PPI network analysis indicated that CDT1 and its similar genes were primarily related to the cell cycle, DNA replication, and the MCM complex. The MCM protein family, including MCM2–10, is reportedly responsible for modulating the cell cycle and DNA replication in eukaryotes (32). Further, overexpression of CDT1 and MCMs has been previously reported in several human cancers (29, 32). Our PPI network analysis identified a close correlation between CDT1 and MCM family genes in HCC. Further analysis revealed their transcriptional levels were highly correlated in HCC. In addition, mRNA levels of MCMs, except MCM9, were markedly increased in HCC samples relative to normal samples, and their high expression predicted poor HCC prognosis. Combined, these results indicate a close relationship between MCMs and CDT1, as well as their expression and prognostic significance in HCC patients, which suggests a potential mechanism through which CDT1 and MCMs cooperate to promote the occurrence and development of HCC.

An increasing body of evidence supports the hypothesis that immune cell infiltration influences the occurrence and progression of cancer, which adversely affects clinical prognosis and immunotherapy effectiveness (36). The relationship between CDT1 mRNA level and the degree of immune cell infiltration in HCC was another significant finding of this study. CDT1 expression was significantly associated with the abundance of activated dendritic cells, T follicular helper cells, neutrophils, dendritic cells, cytotoxic cells, mast cells, and especially Th2 cells. Th cells are important immune regulatory cells in the body and the Th1/Th2 ratio is in dynamic equilibrium under normal conditions (37). When the secretion of Th2 cytokines increases in patients with malignant tumors, Th1/Th2 drift will occur, resulting in Th1/Th2 imbalance (38). Many tumors, including lung cancer, liver cancer, and gastric cancer, have a Th1/Th2 balance shift often dominated by Th2 cells in the body, which may be related to the immune escape of tumors (39). Consistent with the above information, we found that CDT1 expression was positively associated with Th2 cell infiltration in HCC. Furthermore, the Cox proportional hazard model revealed that B cells, CD8+ T cells, CD4+ T cells, macrophages, and dendritic cells were explicitly associated with undesirable clinical outcomes of HCC patients.

Finally, we investigated the impact of CDT1 expression on the malignant phenotype of HCC cells *in vitro*. CDT1 knockdown significantly inhibited, whereas overexpression significantly promoted the proliferation, migration, and invasion of LM3 and Hep3B cells. These results suggest that CDT1 may play an important role in facilitating the development of HCC cells, although the exact downstream mechanism underlying this effect remains to be determined.

Although this study revealed the potential significance and possible mechanism of CDT1 in the occurrence and development of HCC, there were some limitations. First, the functional assessment of CDT1 was based on an *in vitro* model and was not confirmed *in vivo*, and needs to be further explored in future studies. Second, the expression of CDT1 and its prognostic significance need to be verified in clinical samples, as the use of public data sets leads to some errors. Finally, although this study demonstrated that CDT1 plays a role in regulating the cell cycle and influencing immune infiltration, the underlying molecular mechanisms and signaling pathways have not been explored. We will conduct future studies to elucidate the mechanism of CDT1 in HCC.

## Conclusion

In conclusion, we comprehensively and systematically evaluated the expression patterns, prognostic and diagnostic value, and potential mechanisms of CDT1 in the occurrence and development of HCC. Our results provide novel insight to help identify new prognostic biomarkers and therapeutic targets, which may assist clinicians to more accurately predict the survival of HCC patients and inform their treatment decisions.

## Data Availability Statement

Publicly available datasets were analyzed in this study. This data can be found here: https://portal.gdc.cancer.gov/ (The Cancer Genome Atlas (TCGA) program).

## Author Contributions

CC and TC developed the idea and designed the research. YZ, XH, WH, SY, and HQ analyzed the data. CC wrote the draft of the manuscript. TC supervised the project. All authors contributed to the article and approved the submitted version.

## Funding

This research was funded by the General project of Chongqing Natural Science Foundation of China (No. cstc2020jcyj-msxmX0688).

## Conflict of Interest

The authors declare that the research was conducted in the absence of any commercial or financial relationships that could be construed as a potential conflict of interest.

## Publisher’s Note

All claims expressed in this article are solely those of the authors and do not necessarily represent those of their affiliated organizations, or those of the publisher, the editors and the reviewers. Any product that may be evaluated in this article, or claim that may be made by its manufacturer, is not guaranteed or endorsed by the publisher.

## Supplementary Material

Supplementary Figure 1 Results of enrichment analysis of CDT1 and its co-expressed genes. **(A)** The co-expression network of CDT1 gene. **(B, C)** GO and KEGG enrichment results of CDT1 and its co-expressed genes.

Supplementary Figure 2 Interaction network of CDT1 and its most co-expressed genes on STRING.

Supplementary Figure 3 Expression, prognosis of MCM9 and its correlation with CDT1 in HCC. **(A)** Spearman correlation analysis of the correlation between MCM9 and CDT1 at the transcriptional level. **(B)** MCM9 expression between HCC tissues and normal liver tissues. **(C)** Survival analysis of MCM9 in HCC. **p* < 0.05, ***p* < 0.01, ****p* < 0.001.

## Abbreviations

AUC, area under the ROC curve; CCK‐8, Cell Counting Kit‐8; CCLE, The Cancer Cell Line Encyclopedia; CDC6, cell division cycle 6; CDT1, cdc10-dependent transcript 2; CI, confidence interval; DEGs, Differentially expressed genes; DSS, disease-specific survival; FC, fold change; GO, gene ontology; GSEA, Gene set enrichment analysis; GTEx, Genotype-Tissue Expression; HCC, Hepatocellular carcinoma; HPA, Human Protein Atlas; KEGG, Kyoto Encyclopedia of Genes and Genomes; LIHC, liver hepatocellular carcinoma; MCMs, minichromosome maintenance proteins; ORC, origin recognition complex; OS, overall survival; PFS, progression-free survival; PPI, protein-protein interaction; pre-RC, pre-replicating complex; RFS recurrence-free survival; ROC, Receiver operating characteristic; TCGA, the cancer genome atlas; Th, T helper; TILs, tumor-infiltrating lymphocytes.
