# Corrigendum: CDT1 is a Novel Prognostic and Predictive Biomarkers for Hepatocellular Carcinoma

**DOI:** 10.3389/fonc.2021.801970

**Published:** 2021-11-09

**Authors:** Chenhui Cai, Ying Zhang, Xu Hu, Wenhui Hu, Sizhen Yang, Hao Qiu, Tongwei Chu

**Affiliations:** ^1^ Department of Orthopedics, Xinqiao Hospital, Third Military Medical University (Army Medical University), Chongqing, China; ^2^ Department of Biomedical Materials Science, Third Military Medical University (Army Medical University), Chongqing, China

**Keywords:** CDT1, liver hepatocellular carcinoma, prognostic value, immune infiltration, bioinformatics analysis

In the original article, there was a mistake in **Figure 11** as published. In **Figures 11D, E**, we put the wrong pictures of **wound healing assay in NC group at 0 h of both conditions**. We made a mistake when we dragged the original figure into the AI software. We re-examined the original experiment notes and confirmed that this omission did not affect the statistical results and conclusions. The corrected **Figure 11** appears below.

**Figure 11:**
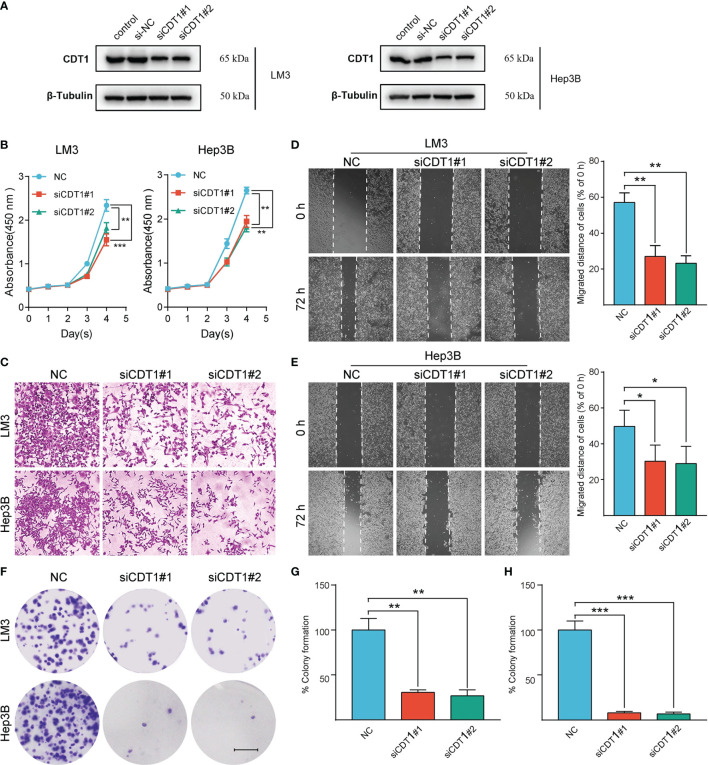
Silencing of CDT1 inhibited the proliferation, migration and invasion of HCC cells. **(A)** Western blot detection of CDT1 expression after knockdown of CDT1 in liver cancer cells. **(B)** The effect of CDT1 knockdown on cell viability at 24, 48, 72 and 96h after seeding in plates was measured by CCK-8 assay. **(C–E)** Transwell analysis and wound healing assay reflected the migration ability of LM3 and Hep3B cell lines. **(F)** Images of the colony formation assay after knockdown of CDT1 in HCC cells **(G, H)** Relative quantification of the colony areas is shown. Scale bars in **(F)** equal 5mm. Data are expressed as means ± SD of three independent experiments. *p < 0.05, **p < 0.01, ***p < 0.001.

The authors apologize for this error and state that this does not change the scientific conclusions of the article in any way. The original article has been updated.

## Publisher’s Note

All claims expressed in this article are solely those of the authors and do not necessarily represent those of their affiliated organizations, or those of the publisher, the editors and the reviewers. Any product that may be evaluated in this article, or claim that may be made by its manufacturer, is not guaranteed or endorsed by the publisher.

