# Supplementary figures and images for: CDT1 Is a Novel Prognostic and Predictive Biomarkers for Hepatocellular Carcinoma
Source: Front Oncol. 2021 Sep 24;11:721644. doi: 10.3389/fonc.2021.721644 (PMC8497762; doi:10.3389/fonc.2021.721644)

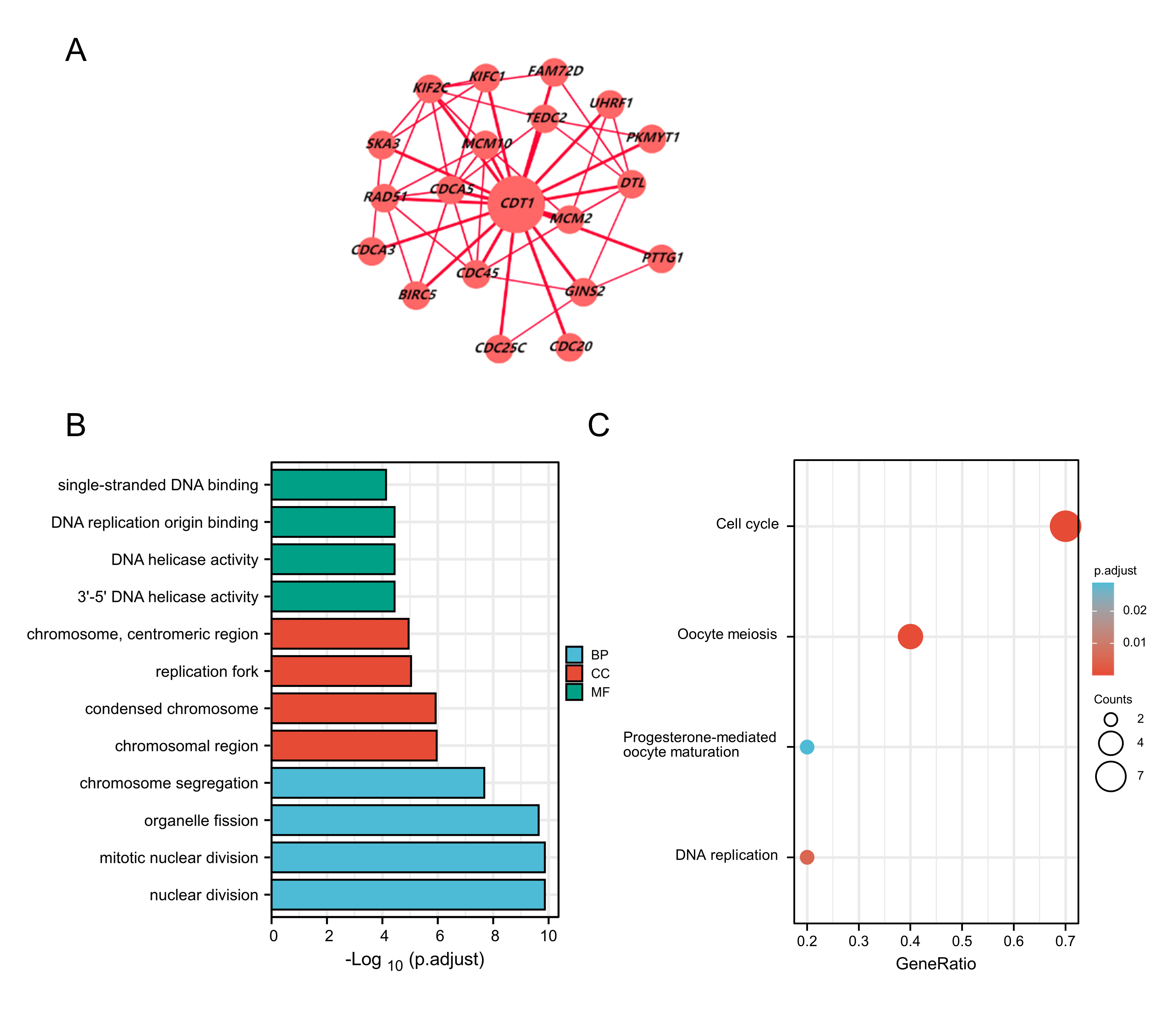

Supplement: Supplementary Figure 1 — Results of enrichment analysis of CDT1 and its co-expressed genes. (A) The co-expression network of CDT1 gene. (B, C) GO and KEGG enrichment results of CDT1 and its co-expressed genes. [file Image_1.tif]

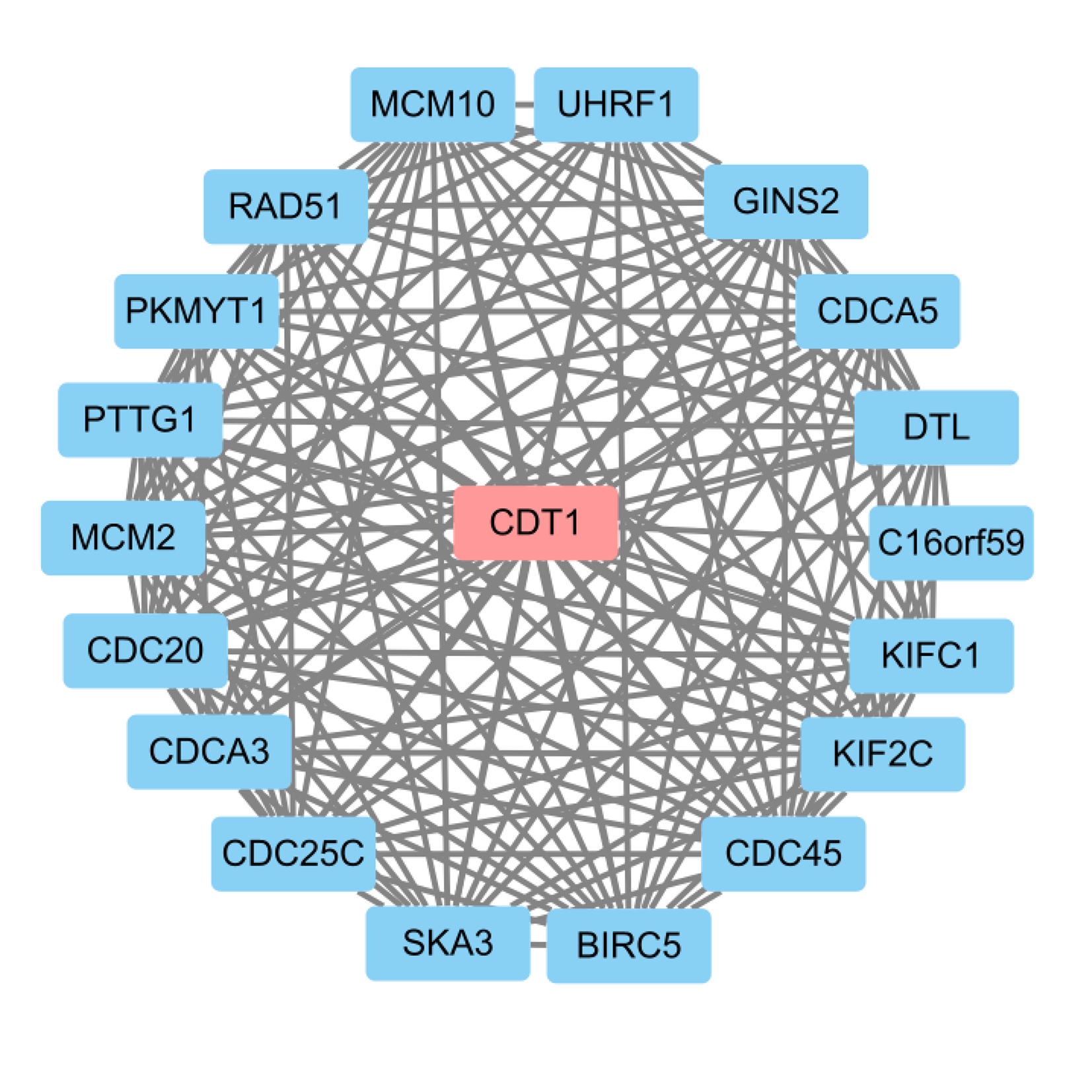

Supplement: Supplementary Figure 2 — Interaction network of CDT1 and its most co-expressed genes on STRING. [file Image_2.tif]

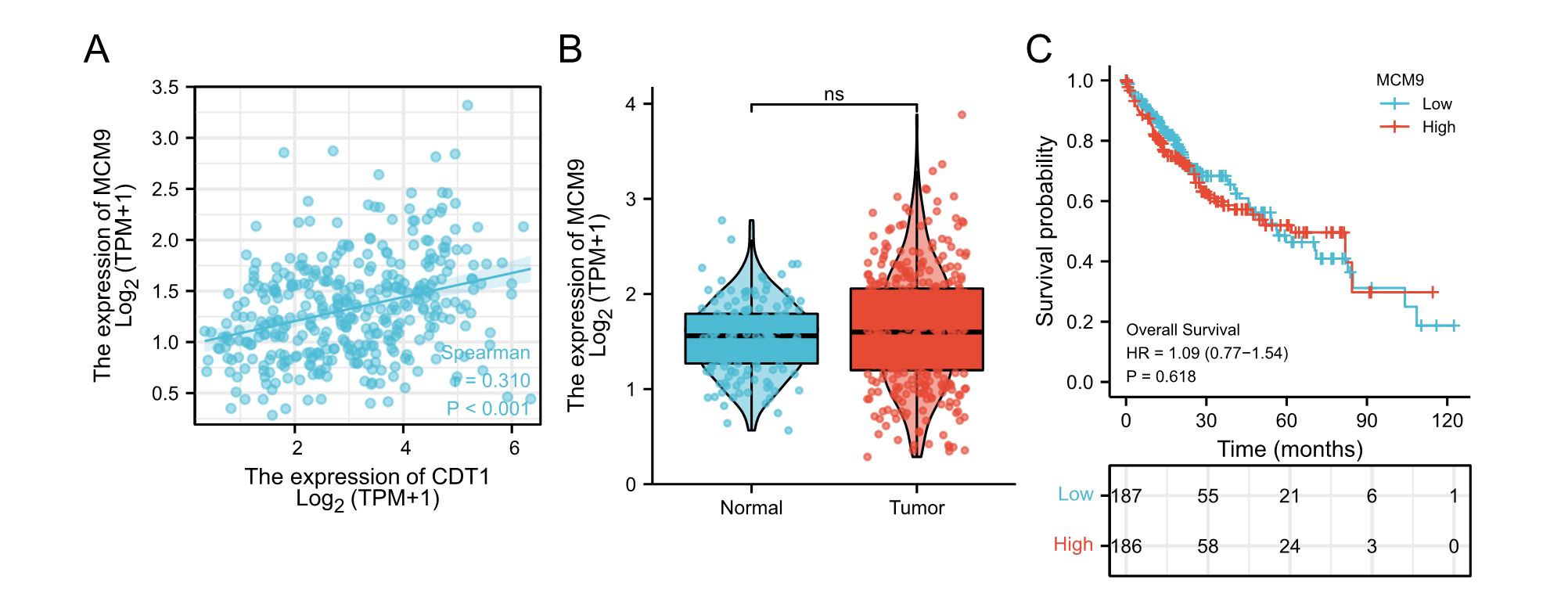

Supplement: Supplementary Figure 3 — Expression, prognosis of MCM9 and its correlation with CDT1 in HCC. (A) Spearman correlation analysis of the correlation between MCM9 and CDT1 at the transcriptional level. (B) MCM9 expression between HCC tissues and normal liver tissues. (C) Survival analysis of MCM9 in HCC. *p < 0.05, **p < 0.01, ***p < 0.001. [file Image_3.tif]
