# Supplementary material for: CDT1 Is a Novel Prognostic and Predictive Biomarkers for Hepatocellular Carcinoma
Source: Front Oncol. 2021 Sep 24;11:721644. doi: 10.3389/fonc.2021.721644 (PMC8497762; doi:10.3389/fonc.2021.721644)
Supplement: Supplementary file 4 [file Table_1.docx]

**Supplymentary Table 1.** The correlation between CDT1 and clinicopathological parameters in HCC.

| **Characteristic** | **Low expression of CDT1** | **High expression of CDT1** | ***p*** |
| --- | --- | --- | --- |
| **N** | 187 | 187 |  |
| **Age, n (%)** |  |  | 0.196 |
| <=60 | 82 (22%) | 95 (25.5%) |  |
| >60 | 105 (28.2%) | 91 (24.4%) |  |
| **Gender, n (%)** |  |  | **0.047** |
| Female | 51 (13.6%) | 70 (18.7%) |  |
| Male | 136 (36.4%) | 117 (31.3%) |  |
| **Race, n (%)** |  |  | **0.006** |
| Asian | 64 (17.7%) | 96 (26.5%) |  |
| Black or African American | 10 (2.8%) | 7 (1.9%) |  |
| White | 105 (29%) | 80 (22.1%) |  |
| **Pathologic stage, n (%)** |  |  | **< 0.001** |
| Stage I | 104 (29.7%) | 69 (19.7%) |  |
| Stage II | 37 (10.6%) | 50 (14.3%) |  |
| Stage III | 29 (8.3%) | 56 (16%) |  |
| Stage IV | 4 (1.1%) | 1 (0.3%) |  |
| **T stage, n (%)** |  |  | **< 0.001** |
| T1 | 111 (29.9%) | 72 (19.4%) |  |
| T2 | 39 (10.5%) | 56 (15.1%) |  |
| T3 | 28 (7.5%) | 52 (14%) |  |
| T4 | 6 (1.6%) | 7 (1.9%) |  |
| **N stage, n (%)** |  |  | 1.000 |
| N0 | 120 (46.5%) | 134 (51.9%) |  |
| N1 | 2 (0.8%) | 2 (0.8%) |  |
| **M stage, n (%)** |  |  | 0.339 |
| M0 | 123 (45.2%) | 145 (53.3%) |  |
| M1 | 3 (1.1%) | 1 (0.4%) |  |
| **Residual tumor, n (%)** |  |  | 0.462 |
| R0 | 168 (48.7%) | 159 (46.1%) |  |
| R1 | 7 (2%) | 10 (2.9%) |  |
| R2 | 1 (0.3%) | 0 (0%) |  |
| **AFP (ng/ml), n** (%) |  |  | **< 0.001** |
| <=400 | 126 (45%) | 89 (31.8%) |  |
| >400 | 19 (6.8%) | 46 (16.4%) |  |
| **Child-Pugh grade, n (%)** |  |  | 0.567 |
| A | 117 (48.5%) | 102 (42.3%) |  |
| B | 13 (5.4%) | 8 (3.3%) |  |
| C | 1 (0.4%) | 0 (0%) |  |
| **Fibrosis ishak score, n (%)** |  |  | 0.895 |
| 0 | 44 (20.5%) | 31 (14.4%) |  |
| 1/2 | 16 (7.4%) | 15 (7%) |  |
| 3/4 | 17 (7.9%) | 11 (5.1%) |  |
| 5/6 | 47 (21.9%) | 34 (15.8%) |  |
| **Vascular invasion, n (%)** |  |  | 0.104 |
| No | 114 (35.8%) | 94 (29.6%) |  |
| Yes | 49 (15.4%) | 61 (19.2%) |  |
